# Methods to normalize surface electromyography in respiratory muscles: Is it similar between amyotrophic lateral sclerosis and healthy people?

**DOI:** 10.1371/journal.pone.0315846

**Published:** 2024-12-20

**Authors:** Thiago Bezerra Wanderley e Lima, Jessica Danielle Medeiros da Fonseca, Ana Aline Marcelino da Silva, Rayane Grayce da Silva Vieira, Dayane Montemezzo, Matias Otto-Yáñez, Rodrigo Torres-Castro, Mário Emílio Teixeira Dourado Júnior, Vanessa Regiane Resqueti, Guilherme Augusto de Freitas Fregonezi

**Affiliations:** 1 PneumoCardioVascular Lab/Hospital Universitário Onofre Lopes (HUOL), Empresa Brasileira de Serviços Hospitalares (EBSERH), Universidade Federal do Rio Grande do Norte, Natal, Rio Grande do Norte, Brazil; 2 Laboratório de Inovação Tecnológica em Reabilitação, Departamento de Fisioterapia, Universidade Federal do Rio Grande do Norte, Natal, Rio Grande do Norte, Brazil; 3 Centro de Ciências da Saúde e do Esporte, Universidade do Estado de Santa Catarina (UDESC), Florianópolis, Santa Catarina, Brazil; 4 Escuela de Kinesiología, Universidad Autónoma de Chile, Santiago de Chile, Chile; 5 Department of Physical Therapy, University of Chile, Santiago de Chile, Chile; 6 Department of Integrated Medicine, Onofre Lopes University Hospital, Federal University of Rio Grande do Norte, Natal, Brazil; Università degli Studi di Milano: Universita degli Studi di Milano, ITALY

## Abstract

The normalization process is important to determine the best approach for normalizing electromyographic signals from respiratory muscles in healthy subjects and those with ALS. The aim of this study is to compare different methods of normalizing the sEMG signal of respiratory muscles in both healthy subjects and those with Amyotrophic Lateral Sclerosis (ALS). This cross-sectional study was conducted in 67 subjects (50 healthy and 17 with ALS). The electrical activity of the sternocleidomastoid (SCM), scalene (ESC), diaphragm (DIA), parasternal (PS), external intercostal (EI), external oblique (EO), and rectus abdominal (RA) muscles were analyzed during maximal inspiratory pressure maneuvers (MIP), maximal nasal inspiratory pressure (SNIP), maximal expiratory pressure (MEP), and maximal voluntary isometric contraction of SCM and ESC (MVIC_SCM/ESC_) and RA (MVIC_RA_) using surface electromyography (sEMG). In the healthy group, inspiratory and expiratory muscles displayed higher electrical activity during MVIC_SCM/ESC_ and MIVC_RA_ maneuvers, respectively (p<0.05). In the ALS group, inspiratory muscle activity was higher during the SNIP maneuver, while expiratory muscles showed higher activity during MVICRA (p<0.05). Based on the findings, it can be concluded that the MVIC resulted in greater inspiratory muscle activity, being the ideal method of normalization for inspiratory and expiratory muscles in healthy subjects. In ALS patients, the SNIP maneuver resulted in greater inspiratory muscle activity, while MVIC resulted in greater muscle activity in expiratory muscles.

## 1. Introduction

Surface electromyography (sEMG) is one of the methods used to assess an individual’s muscle electrical activity, whether at rest or during different activities [1, 2]. The sEMG can be employed to evaluate both skeletal and respiratory muscles in healthy individuals and those with different diseases, enabling an analysis across different times, individuals, and situations [3, 4].

The electromyographic signal is influenced by intrinsic factors related to the muscle’s physiological, anatomical, and biochemical characteristics and by extrinsic factors such as electrode placement, configuration, and skin preparation [5, 6]. Therefore, it is essential to seek methods that aim to mitigate the interference of these factors, making the interpretation of muscle activity more reliable.

Normalization is one of the approaches used for this purpose. Through normalization, a method is used to obtain an electromyographic value considered maximal, which serves as a reference for the signals collected during specific tasks [7, 8]. Therefore, for normalization, investigations involving sEMG must identify the measure of greatest muscle activation, thus producing the best signal, and establish it as a reference. The normalization process is especially important when the goal is to compare the sEMG signals between different individuals and moments [1, 9, 10]. Furthermore, the importance of normalising the sEMG signal in different muscle types has already been described in the literature [8, 11]. Each muscle may have an optimal method for performing normalization, making it important to analyze the best normalization method.

Electromyography has been applied in various situations and conditions affecting muscle activity. Amyotrophic Lateral Sclerosis (ALS) is a progressive neurodegenerative disease characterized by widespread muscle weakness. One of the leading causes of death in these patients is respiratory muscle weakness and, consequently, ventilatory failure [12, 13]. The sEMG becomes a valuable tool for monitoring respiratory muscles in ALS patients, enabling an understanding of muscle recruitment patterns and potential muscular dysfunctions in these individuals.

In the literature, there are different methods for normalization, including maximal voluntary isometric contraction (MVIC), peak dynamic or isometric activities, and the peak of the activity itself [4, 13, 14]. The MVIC-based normalization method has been recognized as the most used and exhibits good reproducibility [4]. However, there is no concrete evidence determining the superiority of these methods [15]. It is currently known that the amplitude of the sEMG signal is dependent on the parameters and method used to normalise the signal [16]. Therefore, it is important to determine the best approach for normalizing electromyographic signals from respiratory muscles in healthy subjects and those with ALS, to reduce potential interferences and obtain better results. Accordingly, this study aims to compare different methods of normalizing sEMG signals from respiratory muscles in individuals with ALS and healthy subjects. Our hypothesis is that in healthy subjects, the MVIC is the best normalization method for inspiratory and expiratory muscles. For the ALS group, our hypothesis is that the SNIP is the best normalization method for inspiratory muscles due to its ease of execution, and the MVIC is the best normalization method for expiratory muscles.

## 2. Methods

### 2.1 Participants

This was a cross-sectional analytical study conducted at the PneumoCardiovascular Lab, Federal University of Rio Grande do Norte (UFRN). The study received ethical approval from research ethics committee of the Hospital Universitário Onofre Lopes (HUOL/EBSERH—Brazil) under number 3.127.064. Participants were collected between September 4, 2019 and March 12, 2020 and all participants signed the informed consent. In the healthy group, participants were recruited in the comunnity by direct invitation, adhering to the following inclusion criteria: age between 19 and 30 years, a body mass index (BMI) between 18 and 25 kg/m², no history of smoking, no respiratory, cardiac, or neuromuscular diseases, and those with normal spirometry [17]. The ALS group included individuals with a definitive ALS diagnosis, as determined by a neurologist follow the criterias: signs of lower motor neuron (LMN) degeneration by clinical, electrophysiological or neuropathologic examination, signs of upper motor neuron (UMN) degeneration by clinical examination, and progressive spread of signs within a region or to other regions [18]; who did not have associated cardiovascular, pulmonary, or other neurological diseases, severe bulbar weakness preventing the completion of maneuvers, or tracheostomy or gastrostomy. Those who did not understand or could not perform the required maneuvers were excluded from the study.

### 2.2 Study design

**Fig 1:**
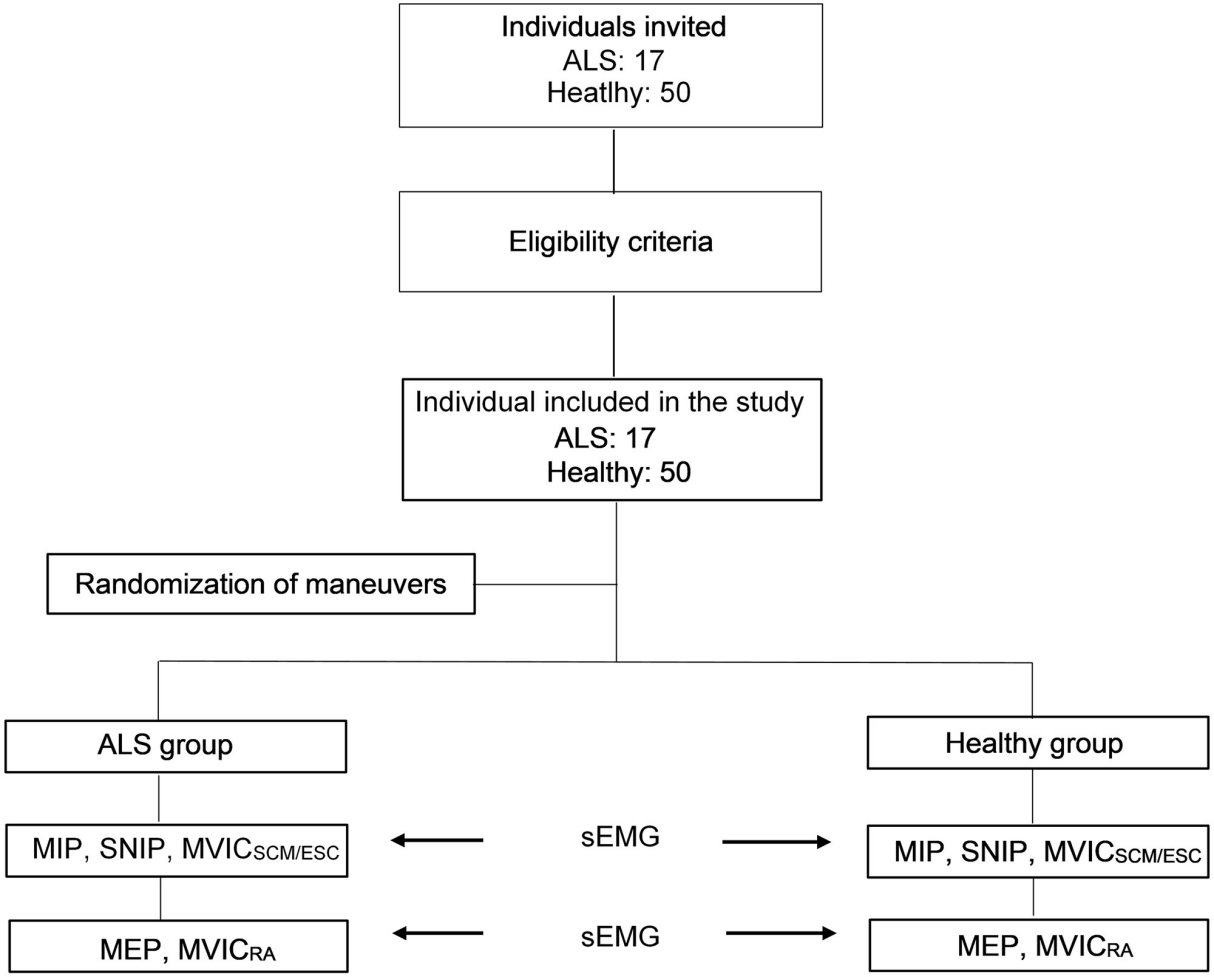
Study design. ALS: Amyotrophic lateral sclerosis; MIP: maximum inspiratory pressure; SNIP: Inspiratory nasal pressure; MVIC_SCM/ESC_: maximum voluntary contraction of sternocleidomastoid and scalene; MEP: maximum expiratory pressure; MVIC_RA_: Maximum voluntary isometric contraction of the rectus abdominis; sEMG: Surface Electromyography.

Two methods of normalizing sEMG signals of respiratory muscles was compared, using maximal respiratory maneuvers as mouth and nasal maximal respiratory pressure (MIP, MEP and SNIP) and Maximal Voluntary Isometric Contraction methods. The evaluations occurred in three stages: 1) clinical assessment and pulmonary function testing; 2) performance of inspiratory maneuvers: maximal inspiratory pressure (MIP), maximal nasal inspiratory pressure (SNIP), and maximal voluntary isometric contraction of sternocleidomastoid and scalene (MVIC_SCM/ESC_); 3) performance of expiratory maneuvers: maximal expiratory pressure (MEP) and maximal voluntary isometric contraction of rectus abdominal (MVIC_RA_). Concurrent with the assessment of stages 2 and 3, muscle activity was measured using the sEMG. The order of stages 2 and 3 was determined by external research with a simple randomization using sealed envelopes. Fig 1 illustrates the study design.

### 2.3 Pulmonary function

The spirometry was conducted using the KoKo DigiDoser spirometer (Longmont, USA). Technical procedures and acceptability criteria followed the American Thoracic Society/European Respiratory Society (ATS/ERS) guidelines [19]. Reference values for the Brazilian population, as published in the Brazilian Guidelines for pulmonary function tests, were adopted [20].

### 2.4 Respiratory muscle strength

The respiratory muscle strength was determined by measuring MIP, MEP, and SNIP using a digital manovacuometer (NEPEB-LabCare / UFMG, Belo Horizonte, MG, Brazil). Assessments adhered to ERS acceptability and reproducibility criteria [15]. Reference values previously published by Neder et al. [21] were used for MIP and MEP, while reference values from Araújo et al. [22] were used for SNIP.

### 2.5 Maximal voluntary isometric contraction

For MVIC, participants were instructed to perform three maximum sustained contractions lasting 5 seconds, with a 1-minute rest between each maneuver [4, 23, 24]. Verbal encouragement was provided during each maneuver to ensure maximal effort. MVIC of the SCM and ESC muscles (MVIC_SCM/ESC_) was performed with participants in a supine position, with legs and arms extended by their sides. They were then asked to perform a contralateral (left) neck rotation while the assessor applied resistance to the temporal region [23]. For MVIC_RA_, participants remained supine on the examination table, with knees flexed to 90 degrees, arms extended by their sides. Participants were then asked to perform a trunk flexion, lifting the scapulae from the table, while the assessor applied resistance to the shoulder region [24].

### 2.6 Surface electromyography

The sEMG was performed according to the SENIAM recommendations [25]. Electromyographic signals were acquired using the TeleMyo DTS Desk Receiver^®^ (Noraxon U.S.A. Inc., Scottsdale, USA) with four wireless Clinical DTS sensors (Noraxon U.S.A. Inc., Scottsdale, USA). The signal was sampled at 1500 Hz, with a 500 Hz low-pass filter, 1000x gain, and a standard mode rejection ratio greater than 100 decibels. Electrodes were placed on the right side of the body to minimize interference from the electrocardiogram. They were positioned in the following muscles: sternocleidomastoid (SCM) in the lower third between the mastoid process and the sternoclavicular joint [26]; scalene (ESC) five centimeters from the sternoclavicular joint and two centimeters above this point [27]; rectus abdominal (RA) 4 cm from the umbilical scar [23]; diaphragm (DIA) in the seventh or eighth intercostal space, according to the best signal capture between the right axillary line and the midclavicular line [24]; parasternal (PS) in the second intercostal space, 3 cm from the sternum [3]; external oblique (EO) at 50% of the distance between the anterior superior iliac spine and the tip of the eleventh rib [28]; external intercostal (EI) in the second intercostal space, along the midclavicular line [29]. In the healthy group, SCM, ESC, and DIA muscles were analyzed during inspiratory maneuvers, while DIA and RA were analyzed during expiratory maneuvers. In the ALS group, SCM, ESC, PS, and EI were analyzed during inspiratory maneuvers, and EO and RA during expiratory maneuvers.

### 2.7 Analysis of electromyographic signals

The electromyographic signals were stored and analyzed using MR software version 3.8 (Noraxon U.S.A. Inc., Scottsdale, USA). The following filters were applied: removal of the ECG signal, full-wave rectification, smoothing with RMS algorithm and a 50 milliseconds window, and a 20 Hz high-pass Butterworth filter. Raw data were analyzed using RMS (root mean square) and for the MIP, MEP and SNIP maneuvers, the maneuvers that generated the highest pressure value were analyzed, while for MVIC_SCM/ESC_ and MVIC_RA_ an average of the 3 contractions performed was taken. The maneuvers that presented the highest RMS values were considered the best for the normalization process.

### 2.8 Statistical analysis

Sample characteristics were expressed as mean ± standard deviation. The Kolmogorov-Smirnov test was used to assess data normality. The comparison of inspiratory maneuvers (MIP, SNIP, and MVIC_SCM/ESC_) was conducted using the Kruskall-Wallis test with Dunn’s post hoc analysis to identify potential differences. The comparison of expiratory maneuvers (MEP and MVIC_RA_) was performed using the Mann-Whitney test. Statistical analyses were conducted using GraphPad Prism 8^®^ software (GraphPad Software Inc.). The Power (β) and effect size (ES) were estimated and are detailed in the results section of this study, calculated using GPower software version 3.1.9.2 (University of Düsseldorf, Kiel, Germany). Effect size for the comparison of inspiratory maneuvers was calculated using epsilon squared (ɛ^2^) with the following interpretations: small (<0.06), moderate (0.06 to 0.14), and large (>0.14) [30]. For the comparison of expiratory maneuvers, effect size (r) was calculated with the following interpretations: small (<0.10), moderate (0.10 to 0.30), and large (>0.50) [30, 31]. A significance level of p<0.05 was adopted for all statistical analyses with a bilateral distribution.

## 3. Results

A total of 67 subjects participated in the study, seventeen in the ALS group and fifty in the healthy group and. Technical issues with one of the electromyography channels led to the inclusion of only 42 subjects in the analysis of diaphragm muscle electrical activity in the healthy group. The mean age for the ALS group was 52.11 ± 12.9 years and for the healthy group 24.80 ± 4.75 years. Patients in the ALS group had an average score of 37.18 ± 5.82 on the Revised Amyotrophic Lateral Sclerosis Functional Rating Scale (ALSFRS-R), with a minimum score of 30 and a maximum score of 45. The subjects in the healthy group had spirometric normal values. Table 1 presents data related to the sample characteristics.

**Table 1:** Subjects characteristics.

	ALS	Health
Subjects _(n)_	17	50
Age _(years)_	52.11 ± 12.9	24.80 ± 4.75
BMI _(kg/m_^2^_)_	24.61 ± 4.31	22.21 ± 1.65
FVC _(%pred)_	78.47 ± 17.10	92.31 ± 10.91
FEV_1 (%pred)_	78.84 ± 21.37	91.85 ± 10.05
FVC/FEV_1 (%pred)_	81.19 ± 2.04	96.45 ± 9.8
MIP _(%pred)_	69.08 ± 32.21	97.05 ± 30.03
MEP _(%pred)_	77.20 ± 36.52	97.51 ± 25.27

Data presented as mean ± SD. FVC: forced vital capacity; FEV1: forced expiratory volume in the first second; FVC/FEV1: ratio of forced vital capacity to forced expiratory volume in the first second; MIP: maximum inspiratory pressure; MEP: maximum expiratory pressure; m: meters; kg: kilograms; %pred: percentage of predicted.

### 3.1 Inspiratory maneuvers

**Fig 2:**
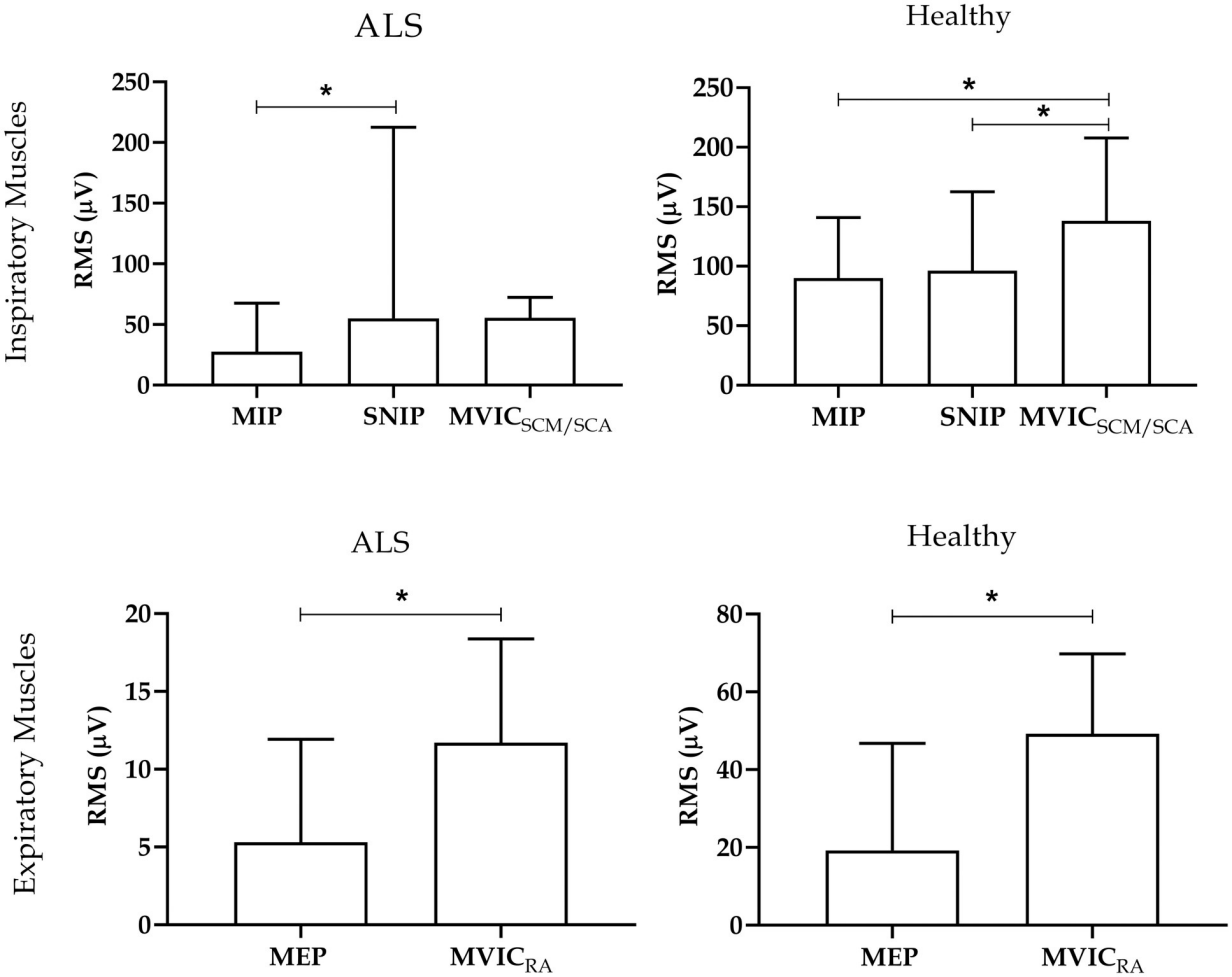
Electromyographic activity of inspiratory and expiratory muscles. Data presented as median and interquartile range. RMS: root mean square; MIP: maximum inspiratory pressure; SNIP: nasal inspiratory pressure; MIVCECOM/ESC: maximum voluntary isometric contraction of sternocleidomastoid and scalene; MEP: maximum expiratory pressure; MIVCRA: maximum voluntary isometric contraction of rectus abdominal. *p<0.05.

The Fig 2 illustrates the electrical activity (RMS) of respiratory muscles during maximal respiratory maneuvers. In the ALS group, inspiratory muscle activity was higher during the SNIP maneuver compared to MIP (p = 0.02). In the healthy group, inspiratory muscles exhibited higher electrical activity during the MVIC_SCM/ESC_ maneuver compared to MIP and SNIP (p<0.05).

**Fig 3:**
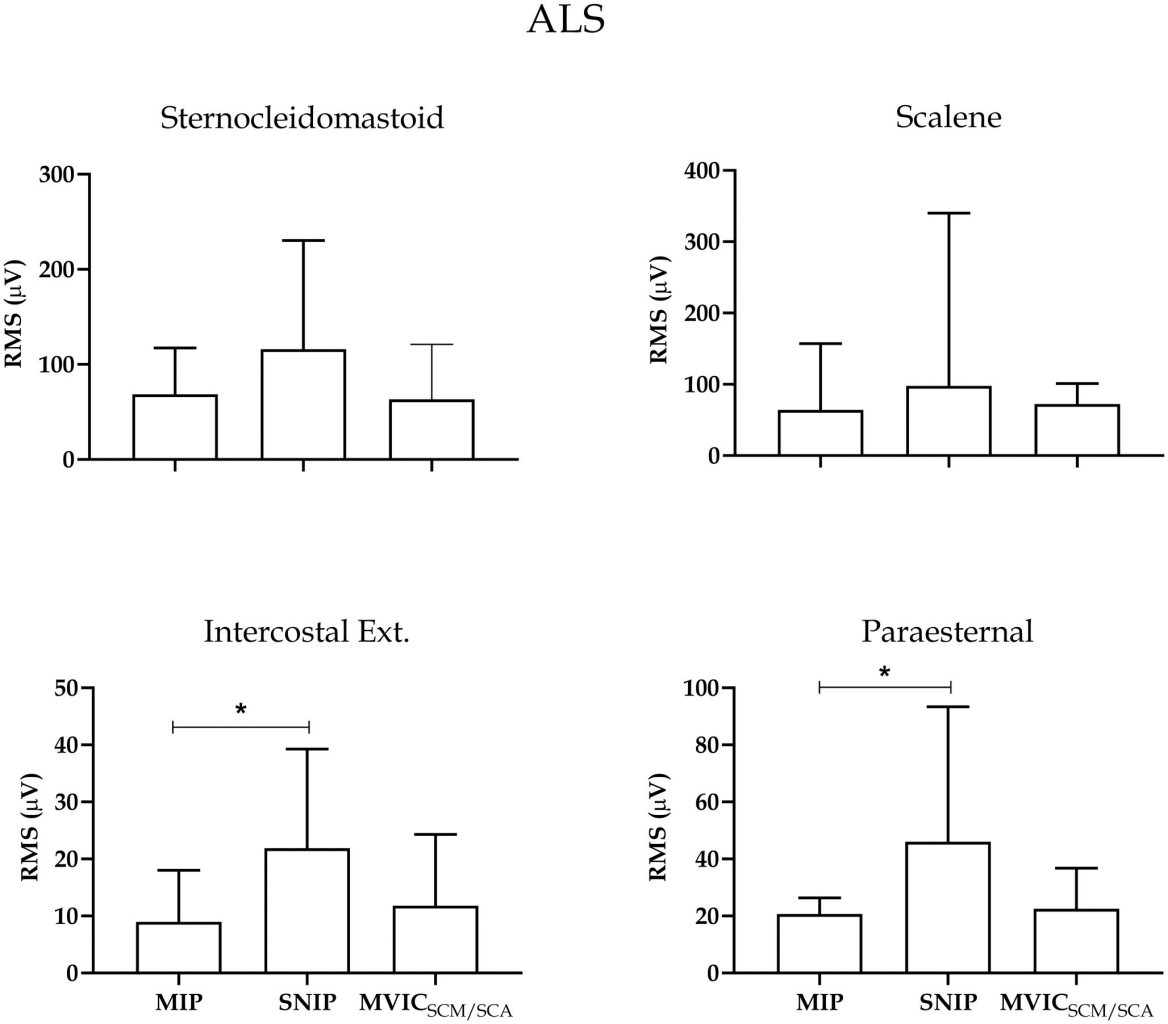
Muscle activity during inspiratory maneuvers in the ALS group. Data presented as median and interquartile range. RMS: root mean square; MIP: maximum inspiratory pressure; SNIP: nasal inspiratory pressure; MIVCECOM/ESC: maximum voluntary isometric contraction of sternocleidomastoid and scalene. *p<0.05.

**Fig 4:**
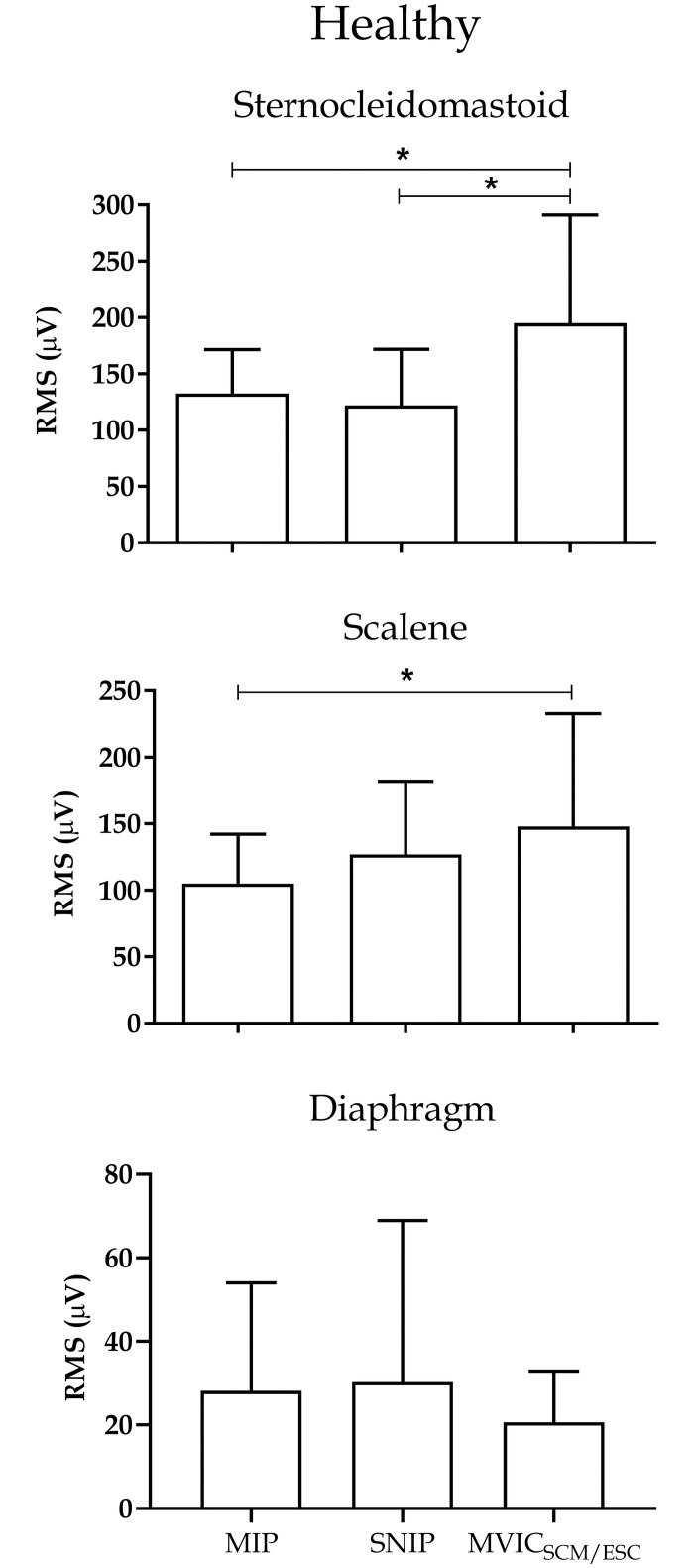
Muscle activity during inspiratory maneuvers in the healthy group. Data presented as median and interquartile range. RMS: root mean square; MIP: maximum inspiratory pressure; SNIP: nasal inspiratory pressure; MIVCECOM/ESC: maximum voluntary isometric contraction of sternocleidomastoid and scalene. *p<0.05.

Figs 3 and 4 provide separate analyses of inspiratory muscles in the ALS and healthy groups, respectively. In the ALS group, the PS and EI muscles exhibited higher electrical activity during the SNIP maneuver compared to MIP (p<0.05). However, the SCM and ESC muscles showed no significant differences between maneuvers (Fig 3). In the healthy group, SCM exhibited higher electrical activity during the MVIC_SCM/ESC_ maneuver compared to MIP and SNIP (p<0.05), and ESC displayed higher activity compared to MIP (p<0.05). The DIA muscle showed no significant differences between maneuvers (Fig 4).

### 3.2 Expiratory maneuvers

**Fig 5:**
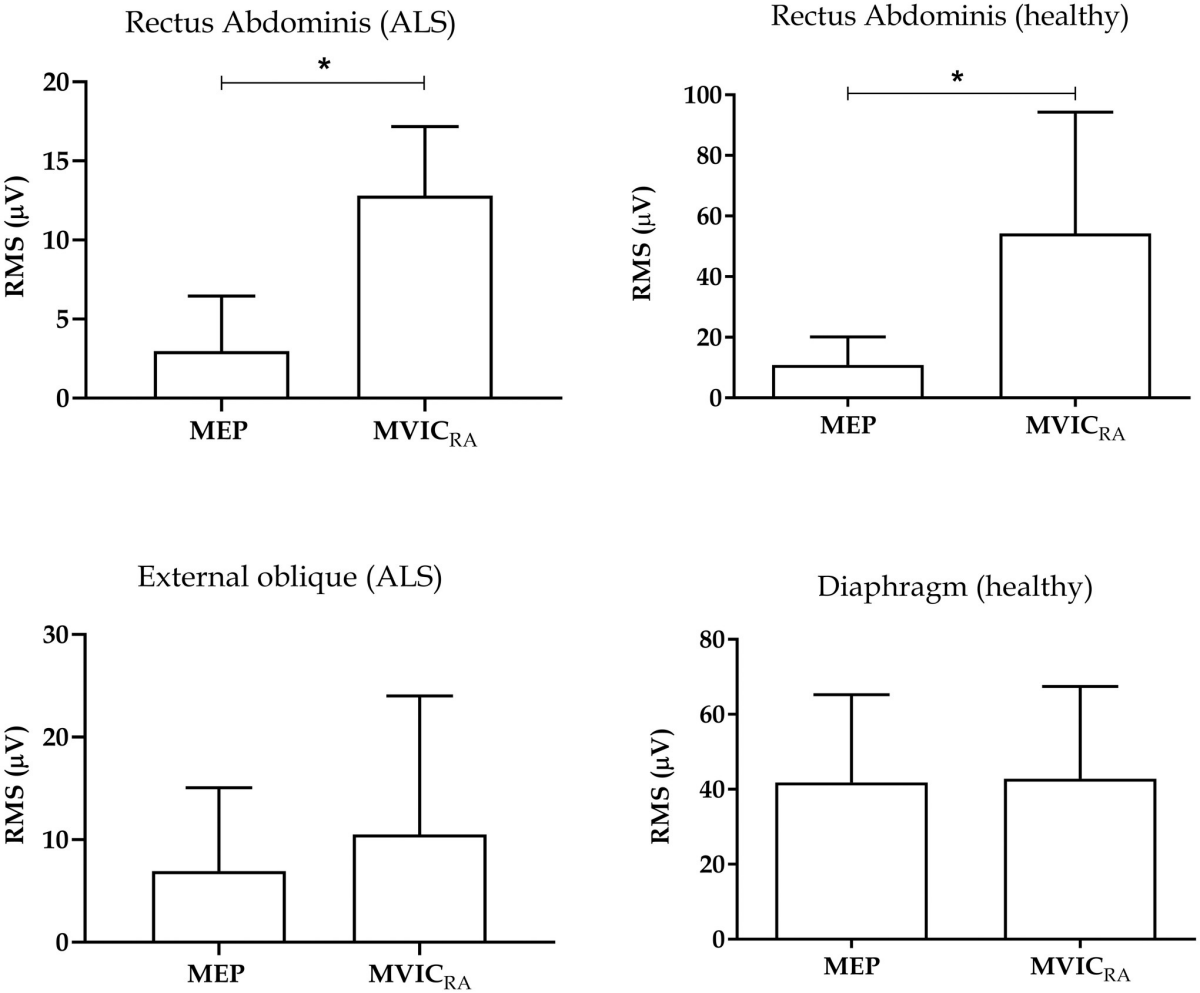
Muscle activity during expiratory maneuvers in the healthy and ALS groups. Data presented as median and interquartile range. RMS: root mean square; MEP: maximum expiratory pressure; MIVCRA: maximum voluntary isometric contraction of rectus abdominal. *p<0.05.

The expiratory muscles showed greater electrical activity during the MVIC_RA_ maneuver compared to MEP in both groups (p<0.05) (Fig 2). Fig 5 presents individual expiratory muscle analyses in the ALS and healthy groups, demonstrating higher RMS values during the MVIC_RA_ maneuver compared to MEP for the RA muscle in both groups (p<0.05). However, the DIA muscle showed no significant differences between maneuvers in the healthy group, and the EO muscle in the ALS group exhibited similar results (Fig 5). The results of the data normality test for inspiratory and expiratory muscles are presented in the supplementary material in S1 and S2 Tables.

### 3.3 Effect size and study power

The Tables 2 and 3 provide post hoc analyses of effect size and study power (1 − β err. Prob.). Both groups showed a moderate effect size for inspiratory muscles considering the inspiratory maneuvers. When analyzing each muscle separately, the EI and PS muscles exhibited a large effect size in the ALS group (ES: 0.18 and ES: 0.35, respectively), while the SCM muscle showed a large effect size in the healthy group (ES: 0.18). In the comparison of expiratory maneuvers, both groups displayed a moderate effect size for expiratory muscles and a large effect size for the RA muscle (ES: 0.52—ALS and 0.67—health).

**Table 2:** Effect size and study power in comparing maneuvers.

Muscle	Maneuvers	Effect size	Power	Effect size	Power
		ALS		Health	
Inspiratory muscles	MIP vs. SNIP vs MVIC_SCM/ESC_	0.06	0.17	0.08	0.27
SCM	MIP vs. SNIP vs MVIC_SCM/ESC_	0.05	0.13	0.18	0.90
ESC	MIP vs. SNIP vs MVIC_SCM/ESC_	0.09	0.33	0.08	0.27
DIA	MIP vs. SNIP vs MVIC_SCM/ESC_	-	-	0.011	0.05
PS	MIP vs. SNIP vs MVIC_SCM/ESC_	0.35	0.99	-	
EI	MIP vs. SNIP vs MVIC_SCM/ESC_	0.18	0.90	-	

SCM: sternocleidomastoid; ESC: scalene; PS: parasternal; DIA: diaphragm; EI: external intercostal; MIP: maximum inspiratory pressure; SNIP: nasal inspiratory pressure; MVICSCM/ESC: maximum voluntary isometric contraction of sternocleidomastoid and scalene; ɛ2: epsilon squared.

**Table 3:** Effect size and study power in comparing maneuvers.

Muscle	Maneuvers	Effect size	Power	Effect size	Power
		ALS		Health	
Expiratory muscles	MEP vs. MVIC_RA_	0.37	0.83	0.39	0.86
RA	MEP vs. MVIC_RA_	0.52	0.98	0.67	0.99
DIA	MEP vs. MVIC_RA_	-	-	0.03	0.05

RA: rectus abdominis; DIA: diaphragm; MEP: maximum expiratory pressure; MVICRA: maximum voluntary isometric contraction of the rectus abdominis.

## 4. Discussion

The aims of this study were to compare two methods of normalizing sEMG signals from respiratory muscles in individuals with ALS and healthy subjects. The main findings in this study revealed that, in healthy subjects, inspiratory and expiratory muscles exhibited greater electrical activity during MVIC_SCM/ESC_ and MVIC_RA_ maneuvers, respectively. Conversely, in ALS patients, inspiratory muscles displayed higher electrical activity during the SNIP maneuver compared only to the MIP maneuver, while expiratory muscles showed increased activity during the MVIC_RA_ maneuver compared to the MEP maneuver. Regarding the analysis of individual muscles, during the maneuvers considered inspiratory, the SCM and ESC muscles exhibited higher electrical activity in the healthy group. In contrast, in the ALS group, the PS and EI muscles demonstrated greater electrical activity during the SNIP maneuver compared to only the MIP. For expiratory maneuvers, the RA muscle exhibited higher electrical activity in both groups during the MVIC_RA_ maneuver. Based on these results, the null hypothesis was rejected.

There are several ways to normalize the respiratory muscles, from maximal maneuvers to simpler maneuvers such as the SNIP [32]. Due to its reproducibility and low variability, the method of normalization through MVIC has been widely used in the literature for various muscle groups and situations [4, 33, 34]. In our study, we observed that the highest electrical activity was found during MVIC maneuvers for the SCM and ESC muscles in inspiratory maneuvers and for the RA muscle in expiratory maneuvers in healthy subjects. This finding aligns with Gandevia et al. [33], who observed in healthy subjects higher electrical activity during neck rotation, neck lateral flexion, and trunk flexion maneuvers compared to respiratory maneuvers in the SCM, ESC, and RA muscles, respectively [33].

The increased electrical activity during MVIC maneuvers in healthy subjects may be related to different muscle recruitment between maneuvers. Motor unit synchronization differs between respiratory and non-respiratory activities [34]. Additionally, the movements performed during MVIC maneuvers are specific to the respective muscle [33]. Supporting our findings, Ito et al. conducted a study assessing the electrical activity of the RA muscle during an expiratory effort maneuver (MEP) and found that the RA had lower electrical activity compared to the external and internal obliques, concluding that the RA is not as recruited during this type of expulsive respiratory maneuver [35].

Patients with ALS exhibit weakness in respiratory muscles, and MIP, MEP, and SNIP maneuvers have been used to evaluate and monitor disease progression. SNIP offers advantages over MIP because it is easier to perform, and many ALS patients have weakness in orofacial muscles, making it difficult to perform the MIP maneuver correctly [36, 37]. This may explain the result of the present study, where inspiratory muscles displayed greater electrical activity during SNIP compared to MIP. Furthermore, orofacial muscle weakness may also explain why the MVIC_RA_ maneuver generated greater electrical activity than MEP, along with the lower activation of the RA compared to other muscles during expulsive respiratory maneuvers, as discussed in the previous paragraph.

sEMG is an important tool for assessing the respiratory muscles in different populations, so the normalization process is essential for better analysis and comparison of these data [38, 39]. In patients with ALS, electromyography plays a key role in diagnosing and monitoring the disease, so establishing the best normalization method contributes to more precise clinical monitoring of deterioration [40]. This information may be useful for professionals who are dedicated to the direct clinical care of patients with ALS as well as for those researchers who are dedicated to the study of electromyographic signals in rapidly progressive neuromuscular diseases. In addition, by identifying the best normalization method for respiratory sEMG signals, a standardised approach to normalizing these signals can be established, facilitating the comparison of clinical and research results.

As a limitation of the study, we have a smaller sample size for the analysis of DIA muscle electrical activity, and the resistance applied during the MVICs of SCM, ESC, and RA was done by manually applying resistance to the subject without using any objective measure of force or pressure, unlike MIP, MEP, and SNIP. Additionally, we used an indirect measure of DIA muscle electrical activity. The strength of our study was the revelation of the best method for normalizing electromyographic signals from respiratory muscles, thus indicating a standard maneuver to be used in future studies aiming to employ the sEMG in various situations and activities.

## 5. Conclusion

In conclusion, in ALS patients, the SNIP maneuver generated higher electrical activity in inspiratory muscles compared only to the MIP, while expiratory muscles showed greater electrical activity during the MVIC maneuver compared to the MEP. In contracts, in healthy subjects, the method of normalization using MVIC was the one in which respiratory muscles exhibited higher electrical activity, both for the set of maneuvers considered inspiratory and expiratory.

## Supporting information

S1 Table Results of data normality test for inspiratory muscles. SCM: sternocleidomastoid; ESC: scalene; PS: parasternal; EI: external intercostal; MIP: maximum inspiratory pressure; SNIP: nasal inspiratory pressure; MVICSCM/ESC: maximum voluntary isometric contraction of sternocleidomastoid and scalene. The normality test used was Kolmogorov-Smirnov and the values presented are the p value for each muscle and maneuver.
(DOCX)

S2 Table Results of data normality test for expiratory muscles. RA: rectus abdominis; DIA: diaphragm; EO: external oblique: MEP: maximum expiratory pressure; MVICRA: maximum voluntary isometric contraction of the rectus abdominis. The normality test used was Kolmogorov-Smirnov and the values presented are the p value for each muscle and maneuver.
(DOCX)
