# Supplementary material for: Methods to normalize surface electromyography in respiratory muscles: Is it similar between amyotrophic lateral sclerosis and healthy people?
Source: PLoS One. 2024 Dec 20;19(12):e0315846. doi: 10.1371/journal.pone.0315846 (PMC11661598; doi:10.1371/journal.pone.0315846)
Supplement: S1 Table — SCM: sternocleidomastoid; ESC: scalene; PS: parasternal; EI: external intercostal; MIP: maximum inspiratory pressure; SNIP: nasal inspiratory pressure; MVICSCM/ESC: maximum voluntary isometric contraction of sternocleidomastoid and scalene. The normality test used was Kolmogorov-Smirnov and the values presented are the p value for each muscle and maneuver. (DOCX) [file pone.0315846.s001.docx]

**S1 Table. Results of data normality test for inspiratory muscles.**

| Muscle | ALS  MIP SNIP MVIC _SCM/ESC_ | | | Health  MIP SNIP MVIC _SCM/ESC_ | | |
| --- | --- | --- | --- | --- | --- | --- |
| Inspiratory muscles | <0.0001 | <0.0001 | 0.0053 | <0.0001 | <0.0015 | 0.0001 |
| SCM | 0.0044 | >0.1000 | 0.0050 | >0.1000 | >0.1000 | 0.0012 |
| ESC | 0.0038 | 0.0023 | 0.0970 | <0.0001 | >0.1000 | 0.0220 |
| DIA | _ | _ | _ | <0.0001 | <0.0001 | 0.0181 |
| PS | >0.1000 | 0.0030 | >0.1000 | _ | _ | _ |
| EI | 0.0636 | 0.0118 | >0.1000 | _ | _ | _ |
|  |  |  |  |  |  |  |

SCM: sternocleidomastoid ; ESC: scalene; PS: parasternal; EI: external intercostal; MIP: maximum inspiratory pressure; SNIP: nasal inspiratory pressure; MVICSCM/ESC: maximum voluntary isometric contraction of sternocleidomastoid and scalene. The normality test used was Kolmogorov-Smirnov and the values presented are the p value for each muscle and manoeuvre.
