# Supplementary material for: Methods to normalize surface electromyography in respiratory muscles: Is it similar between amyotrophic lateral sclerosis and healthy people?
Source: PLoS One. 2024 Dec 20;19(12):e0315846. doi: 10.1371/journal.pone.0315846 (PMC11661598; doi:10.1371/journal.pone.0315846)
Supplement: S2 Table — RA: rectus abdominis; DIA: diaphragm; EO: external oblique: MEP: maximum expiratory pressure; MVICRA: maximum voluntary isometric contraction of the rectus abdominis. The normality test used was Kolmogorov-Smirnov and the values presented are the p value for each muscle and maneuver. (DOCX) [file pone.0315846.s002.docx]

**S2 Table. Results of data normality test for expiratory muscles.**

| Muscle | ALS  MEP MVIC_RA_ | | Health  MEP MVIC_RA_ | |
| --- | --- | --- | --- | --- |
| Expiratory muscles | 0.0002 | 0.0014 | <0.0001 | <0.0001 |
| RA | 0.0078 | 0.0003 | <0.0001 | <0.0001 |
| DIA | _ | _ | 0.0002 | 0.0221 |
| EO | 0.0093 | 0.1030 | _ | _ |

RA: rectus abdominis; DIA: diaphragm; EO: external oblique: MEP: maximum expiratory pressure; MVICRA: maximum voluntary isometric contraction of the rectus abdominis. The normality test used was Kolmogorov-Smirnov and the values presented are the p value for each muscle and manoeuvre.
